# Phytochemical Profile and Antioxidant Activity of Aerial and Underground Parts of *Salvia bulleyana* Diels. Plants

**DOI:** 10.3390/metabo10120497

**Published:** 2020-12-03

**Authors:** Izabela Grzegorczyk-Karolak, Marta Krzemińska, Anna K. Kiss, Monika A. Olszewska, Aleksandra Owczarek

**Affiliations:** 1 Department of Biology and Pharmaceutical Botany, Medical University of Lodz, 90-151 Lodz, Poland; marta.wojciechowska2@stud.umed.lodz.pl; 2 Department of Pharmacognosy and Molecular Basis of Phytotherapy, Medical University of Warsaw, 02-097 Warsaw, Poland; akiss@wum.edu.pl; 3 Department of Pharmacognosy, Medical University of Lodz, 90-151 Lodz, Poland; monika.olszewska@umed.lodz.pl (M.A.O.); aleksandra.owczarek@umed.lodz.pl (A.O.)

**Keywords:** antiradical activity, hydromethanolic extract, lithospermic acid, phenolic profiling, rosmarinic acid, salvianolic acid K, shoots

## Abstract

Plants have been used for medical purposes since ancient times. However, a detailed analysis of their biological properties and their associated active compounds is needed to justify their therapeutic use in modern medicine. The aim of the study was to identify and quantify the phenolics present in hydromethanolic extracts of the roots and shoots of the Chinese *Salvia* species, *Salvia bulleyana*. The qualitative and quantitative analyses were carried out by ultrahigh-performance liquid chromatography with electrospray ionization mass spectrometry detection (UHPLC-PDA-ESI-MS), and high-performance liquid chromatography with photodiode array (HPLC-PDA) detection. The extracts of *S. bulleyana* were also screened for their antioxidant activity using ferric ion (Fe^3+^) reducing antioxidant power (FRAP), 1,1-diphenyl-2-picrylhydrazyl (DPPH), diammonium 2,2′-azino-bis(3-ethylbenzothiazoline-6-sulfonate) cation (ABTS), superoxide radical anion (O_2_•–), and inhibition of lipid peroxidation assays. The *S. bulleyana* extracts were found to contain 38 substances, of which 36 were phenols, with a total level of 14.4 mg/g DW (dry weight) in shoots, and 23.1 mg/g DW in roots. Twenty-eight phenols were polyphenolic acids or their derivatives, the most abundant in shoots being rosmarinic acid, and in roots, salvianolic acid K followed by rosmarinic acid. The other major phenolic acids were caffeic acid, caffeoyl-threonic acids, isomers of lithospermic acid, salvianolic acid F, salvianolic acid B, and yunnaneic acid E. In addition to polyphenolic acids, nine flavonoids were detected in the shoot extract. While both extracts showed significant antioxidant activity, the shoot extract, containing both polyphenolic acids and flavonoids, demonstrated a slightly greater antioxidant potential in some of the anti-radical tests than the roots. However, the root extract proved to be slightly more effective in the lipid peroxidation inhibition test. Thus, *S. bulleyana* was demonstrated as a promising source of antioxidants, and worthy of further more detailed studies.

## 1. Introduction

The prevalence of civilization diseases, such as cancer, circulatory disorders, diabetes mellitus, obesity, and neurodegenerative diseases, is steadily growing worldwide. However, studies have suggested that many natural plant products may counteract the factors causing them. Of these, polyphenol-rich species such as sage (*Salvia*) offer considerable promise [1].

*Salvia* is one of the largest genera in the Lamiaceae family, comprising around 1000 species worldwide. Its representatives have been used in traditional and official medicine, but also as food, spices, and as cosmetics. Over 40 species of *Salvia* have been used in Chinese traditional medicine; twenty of these have been described as Danshen [2]. The Chinese pharmacopeia refers to the root of *S. miltiorrhiza* Bunge, a species endemic to eastern Asia, as Danshen [3]. It has been used in the treatment of coronary heart disease, myocardial infarction, angina pectoris, coronary artery spasm, and for promoting blood circulation [4]. However, other morphologically similar species have been used as folk substitutes for the original Danshen in different regions of China: *S. bowleyana* Dunn, *S. bulleyana* Diels, *S. campanulata* Wall., *S. castanea* Diels, *S. dabieshanensis* J.Q.He, *S. deserta* Schang, *S. digitaloides* Diels, *S. evansiana* Hand.-Mazz., *S. flava* Forrest ex Diels, *S. honania* L.H.Bailey, *S. meiliensis* S.W. Su, *S. paramiltiorrhia* H.W.Li et X.L.Huang, *S. plectranthoides* Griff., *S. przewalski* Maxim.*, S. sinica* Migo*, S. trijuga* Diels*, S. vasta* H.W.Li, and *S. yunnaensis* C.H.Wright [2]. For most of those species, only a little scientific data has been published concerning their phytochemistry and bioactivity. However, preliminary research indicates that many of them do not meet the same quality parameters as Danshen, and should not be used under the same name [5]. Therefore, more detailed knowledge about the phytochemical composition of these plants is needed.

**Figure 1:**
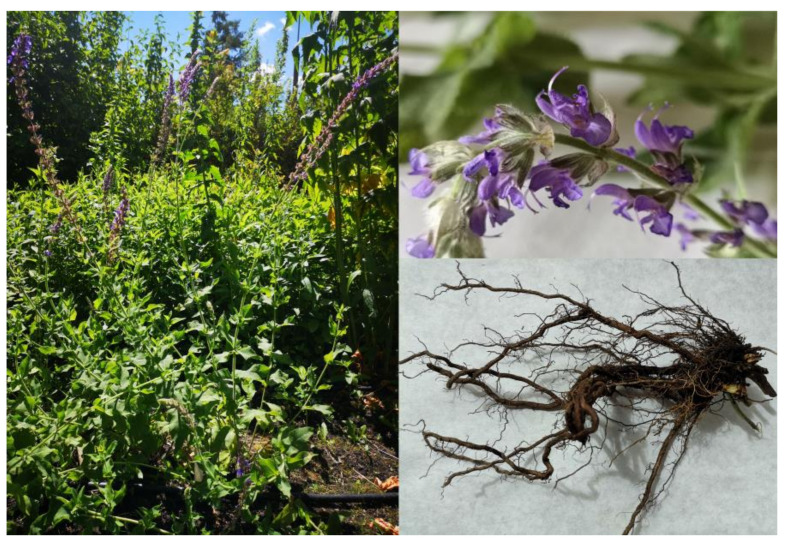
*Salvia bulleyana* plants.

*S. bulleyana*, or Zi Danshen (Figure 1), is a perennial plant growing on hills and valleys in the Yunnan Province. It has brownish roots and ovate to ovate-triangular leaves. The flowers have purple-blue corollas, and grow in whorls, with four flowers to each whorl [4]. According to some reports, *S. bulleyana* roots have been used as Danshen, and their most important and frequent clinical application has been in the treatment of cardiovascular and cerebrovascular diseases [2]. The species’ application in folk medicine is restricted to mountain areas and regions of Yunnan province, where it naturally occurs.

Few phytochemical investigations on *S. bulleyana* have been performed, and they identified two groups of constituents: hydrophilic phenolic acids and lipophilic diterpenoids [2,5,6]. Among the phenolic acids, the roots of *S. bulleyana* contained danshensu, procatechuic aldehyde, salviflaside, salvianolic acid K, salvianolic acid B, caffeic acid, and rosmarinic acid, with the last two also being present in the aerial parts [2,6]. Tanshinones, i.e., abietane diterpenoids such as cryptotanshinone and tanshinone IIA, were detected in the roots of the plant; however, they were present at lower concentrations than in the original Danshen [5]. Therefore, more thorough studies are needed to justify the medical applications of traditional products obtained from *S. bulleyana*. 

The aim of this study was to determine the phytochemical profile of hydromethanolic extracts obtained from both the shoots and roots of *S. bulleyana*. Their antioxidant potential was tested by free radical scavenging (DPPH, ABTS, O_2_*^•^*^−^), metal chelating, and inhibition of lipid peroxidation assays. The obtained results can provide valuable information on the medical potential of the species.

## 2. Results and Discussion

### 2.1. Quantitative Analysis of Analyzed Hydromethanolic Extracts

**Figure 2:**
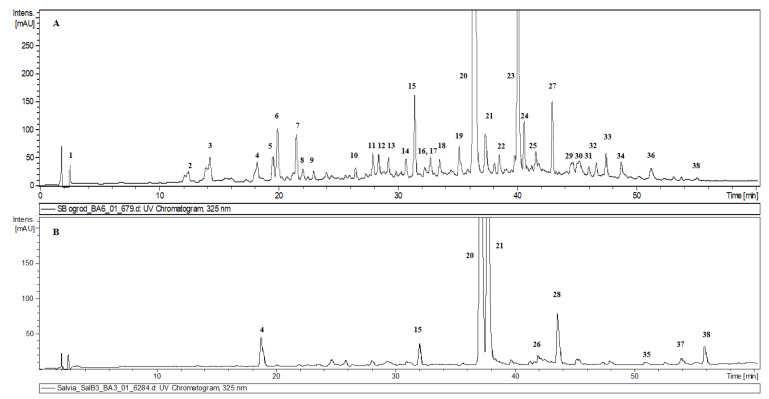
UV chromatograms obtained from the UPLC–MS/MS system recorded at 325 nm of the hydromethanolic extracts from aerial parts (**A**) and roots (**B**) of *S. bulleyana*. Peak numbers correspond to compound numbers in Table 1.

The identification of compounds in hydromethanolic extracts from *S. bulleyana* aerial parts and roots was carried out by ultrahigh-performance liquid chromatography with electrospray ionization mass spectrometry (UHPLC-PDA-ESI-MS^3^). Average extraction yields (% *w*/*w*) were 18.8% for roots, and 23.6% for aerial parts. The chromatograms of both extracts are presented in Figure 2. The analysis led to the detection of 38 compounds, 36 of which (nine in root extract, and 32 in aerial part extract) were fully or tentatively identified as phenolics (Table 1). The identified compounds can be classified into two structural groups: phenolic acids and their derivatives (28 compounds) and flavonoids (nine compounds); however, flavonoids were detected only in the shoot extract. The compounds were identified by comparing the obtained spectra data with those of the authentic standards, and/or those available in the literature [7,8,9,10,11,12,13]. 

#### 2.1.1. Identification of Phenolic Acid Derivatives

All compounds identified in roots, and most of those in shoots were derivatives of phenolic acids. Some detected compounds, such as rosmarinic acid, caffeic acid, and salvianolic acid K, had already been identified in the species [2,6]. Others, such as isomers of lithospermic acid, salvianolic acid F, or yunnaneic acid E, had been found in *Salvia* plants [10,12,14], but had not previously been detected in *S. bulleyana*. The presence of caffeic acid (compound **4**) and rosmarinic acid (compound **20**) was confirmed by using the commercially available standards. Both of these metabolites are commonly found not only in sage species, but also in other species of the Lamiaceae family [10,15].

Peaks **2**, **3,** and **6** had the same deprotonated molecular ion at *m/z* 297 and fragmentation ions at *m/z* 179 (caffeic acid moiety) and 135 (caffeic acid moiety—CO_2_). Such a pattern is characteristic of caffeoyl-threonic acids isolated, for example, from aerial parts of the *Pulmonaria officinalis* plant from the Boraginaceae family, which, like the Lamiaceae family, comprises species rich in caffeic acid derivatives [9]. To our knowledge, caffeoyl-threonic acid isomers were identified in the genus *Salvia* for the first time. 

Peaks **5** and **8** in the MS^2^ spectra exhibited ions characteristic of sinapic acid derivatives, i.e., *m/z* 223 (sinapic acid moiety), and *m/z* 205 (sinapic acid moiety—H_2_O) [16]. Due to the neutral loss of 162 amu, peak **5** was assigned as sinapic acid hexoside. The fragmentation pattern of peak **8** did not allow for its further identification. Derivatives of sinapic acid, found throughout the many Brassicaceae and Rosaceae species [17], are not common for the sage genus, but have also been detected in *S. officinalis* L. [18] and *S. viridis* L. [11].

Peaks **15** (rosmarinic acid hexoside), **25** (dehydrorosmarinic acid), and **28** (methyl rosmarinate) are common rosmarinic acid derivatives present in many other *Salvia* species [6,10,14,21]. On the other hand, peaks **27**, **30,** and **31,** with ion of the deprotonated molecule at *m/z* 727, have not been described in the genus *Salvia* before. The neutral loss of 198 amu (danshensu) and a fragmentary ion at *m*/*z* 359 clearly suggest their being rosmarinic acid derivatives, while the presence of fragmentary ions at *m*/*z* 565 [M−H−162]^−^ and *m*/*z* 521 [M−H−206]^−^ indicates a potential loss of hexose and sinapic acid moieties. The presence of a sinapoyl component was further confirmed by low-intensity product ions at *m*/*z* 223 and 205. A compound that might be consistent with this fragmentation pattern has previously been isolated from *Dracocephalum foetidum* Bunge (Lamiaceae), and identified as 3-(6-synapoyl-*β*-D-glucopyranosyl)-rosmarinic acid [22]. Due to the lack of additional data, the peaks were tentatively identified as rosmarinic acid sinapoyl-hexosides.

The chromatographic behavior and fragmentation pattern of peak **10** was in agreement with the literature data for yunnaneic acid E [12]. Peak **21** was identified as salvianolic acid K, as described previously for *S. bulleyana* [13], and peak **16** as its isomer, found previously in *S. cadmica* Boiss. [20].

Peak **26**, found in the *S. bulleyana* roots, revealed a deprotonated molecular ion [M−H]^−^ at *m/z* 717 and fragmentation ions at *m/z* 519 and 321, consistent with consecutive loss of two danshensu units, which is characteristic of salvianolic acid B. However, the retention time of this compound was different from that obtained for the salvianolic acid B commercial standard. Therefore, this peak was identified as salvianolic acid B isomer. The same identification was tentatively ascribed to peak **36**, present in shoots, with the analogous pseudomolecular ion, but a slightly different fragmentation pattern (*m*/*z* 519, 359, 339).

Peaks **35** and **38** with pseudomolecular ion at *m/z* 313 and a fragmentation pattern very similar to that of salvianolic acid F, however with varying elution times, were tentatively identified as salvianolic acid F isomers. Compounds with analogous characteristics have been previously described in roots of *S. viridis* [19] and aerial parts of *S. blepharochlaena* Hedge & Hub.-Mor., *S. euphratica* Montbret & Aucher., and *S. verticillata* L. [10].

Compounds **23** and **29** exhibited a deprotonated molecule at *m*/*z* 537 that gave a major product ion at *m*/*z* 493 [M−H−CO_2_]. This is a typical fragmentation of lithospermic acid, and a common constituent of original Danshen [25]. However, MS^2^ spectra of both compounds also showed a relatively abundant ion at *m*/*z* 359, which is inconsistent with the structure of lithospermic acid and its MS behavior as described in the literature [23]. Thus, both compounds were tentatively assigned as lithospermic acid isomers. Compound **34,** with a pseudomolecular ion at *m/z* 551 indicating the presence of an additional methyl group, was analogously identified as a methyl derivative of lithospermic acid isomer. 

Compound **22**, detected in the shoots, exhibited a deprotonated molecular ion at *m*/*z* 683, with a major fragmentation ion at *m/z* 485 [M−H−danshensu]^−^, and minor ions at *m/z* 521 [M−H−caffeoyl moiety]^−^ and *m/z* 359 (rosmarinic acid). Although its UV spectrum was also typical of caffeic acid derivatives, its full identification was not possible.

Compounds **24**, **32,** and **38** were classified as phenylethanoids [24]. Compound **24,** with a precursor [M−H] ion at *m*/*z* 651, and its product ions at *m*/*z* 505 [M−H−rhamnose]^−^, 475 [M−H−feruloyl moiety], and 329 [M−H−rhamnose-feruloyl moiety], was identified as martynoside [7]. Compounds **32** and **38** had a very similar fragmentation pattern, with an additional one or two acetyl moieties (42 amu), respectively. Thus, they were characterized as martynoside acetyl derivatives. This is the first report of the presence of phenylethanoids in the organs of *S. bulleyana*. The compound, with data consistent with that of compound **38,** had been detected earlier in hydromethanolic extract from *S. bulleyana* transformed roots, but in that paper it was not identified [13]. Phenylethanoids are not common metabolites of the *Salvia* genus, but they have been detected in the shoots of some species, for example of *S. viridis* [7]. 

#### 2.1.2. Identification of Flavonoids

Nine compounds in the aerial parts of *S. bulleyana* exhibited UV–vis spectra with two maxima characteristic of flavone derivatives [26]. Four of them (**12**, **13**, **14,** and **33**) demonstrated an aglycone fragmentation ion at *m/z* 285 that, taking into account the phytochemistry of the *Salvia* genus, was tentatively identified as luteolin (3′,4′,5,7-tetrahydroxyflavone). The presence of this aglycone was further confirmed after hydrolysis, by comparison with the reference standard. The neutral losses of 176 amu (hexuronic acid moiety), 162 amu (hexose moiety), or 308 amu (rhamnohexose moiety) were observed; this allowed the compounds **12**, **13,** and **14** to be tentatively identified as luteolin *O*-hexoside-*O*-hexuronide, luteolin *O*-rhamnohexoside (most likely rutinoside), and luteolin-*O*-hexuronide, respectively. Compounds with such a structure have not been previously described for *S. bulleyana*, but have been found in extracts from shoots of other sage species [7,8,11]. Peak **33** with a very late elution time presented a pseudomolecular ion at *m/z* 811, and fragment ions at *m/z* 635, 443, and 285. Although it was not possible to identify the compound based on the fragmentation profile; the UV spectrum was typical of a flavonoid, and it was hence categorized as a flavone derivative. Due to the presence of the ion at *m/z* 285, it can be hypothesized that it is also a derivative of luteolin. However, no such derivative of this aglycone is described in the literature.

Peaks **17** and **19,** with pseudomolecular ions at *m/z* 577 and 455, after neutral losses of 308 amu (rhamnohexose moiety) and 176 amu (hexuronic acid moiety), respectively, exhibited the same fragment ion at *m/z* 269. Compounds with a similar fragmentation pattern have been earlier identified as apigenin glycosides in *S. euphratica* [10], *S. officinalis* [15], and *S. verticillata* [14]. However, in the hydrolyzed sample, a apigenin peak was not detected. Thus, the compounds have been tentatively identified as derivatives of trihydroxyflavone.

Peaks **7** and **11** showed pseudomolecular ions at *m/z* 801 and at *m/z* 639, respectively, and a fragmentation ion of the aglycone at *m/z* 301. The aglycone with the same molar mass is characteristic of a quercitin derivative, described earlier for sage extracts [14], however the observed UV spectra showed maxima typical of flavones, not flavanols. The aglycone of these compounds is highly similar to hydroxyluteolin (pentahydroxyflavone), which has an additional hydroxyl group in the 6 or 8 position, and the peaks were identified as, respectively, hydroxyluteolin-*O*-dihexoside-*O*-hexuronide and hydroxyluteolin-*O*-hexoside-*O*-hexuronide. However, hydroxylation of position 6 is more common in flavonoids isolated from *Salvia* species [27]. Those particular derivatives have not been described for *S. bulleyana* or other *Salvia* species, but the presence of this flavonoid and its glycosides has been found in sage extracts [8,11,15,28].

Compound **18** exhibited a pseudomolecular ion at *m/z* 607 and a fragment ion of the aglycone at *m/z* 299 [M–H–308]^−^, and thus it was identified as trihydroxymethoxyflavone rhamnohexoside. A component with an identical fragmentation pattern, detected in *S. officinalis* extract, was identified as hispidulin (4′,5,7-trihydroxy-6-metoxyflavone) rutinoside [8], and in *S. mexicana* L. as chrysoeriol (4′,5,7-trihydroxy-5′-metoxyflavone) rutinoside [12]. However, in our case, the lack of additional information from isolation and NMR studies did not allow for a more precise identification.

#### 2.1.3. Identification of a Non-Phenolic Compound

Peak **1,** detected in the extract from *S. bulleyana* shoots, exhibited a pseudomolecular ion at *m*/*z* 191 which may be quinic acid. The compound was also presented in extracts from other *Salvia* species, including *S. officinalis*, *S. elegans* Vahl., *S. greggii* A. Gray*, S. africana* L., and *S. mexicana* [12,15].

### 2.2. Quantitative Analysis

The analysis of UHPLC-PDA-MS chromatograms indicated that the dominant constituents of the investigated extracts were rosmarinic acid (peak **20**), rosmarinic acid hexoside (peak **15**), salvianolic acid K (peak **21**), salvianolic acid B isomer (peak **26**), methyl rosmarinate (peak **28**), lithospermic acid isomer (peak **23**), and isomer of salvianolic acid F (peak **37**). Thus, for better characterization of the extracts those compounds were assayed quantitatively (Table 2).

The dominant component of the shoots and the second most abundant in the roots was rosmarinic acid, with the contents of 6.07 mg/g DW and 8.34 mg/g DW, respectively. This secondary metabolite was found in similar amounts (7.6 mg/g DW) in the roots of plants grown in field conditions in China by Li et al. [2]. It is a pharmacologically-important bioactive compound common in species of the Lamiceae family. The compound has been found in wild-growing populations of 35 *Salvia* species in China, ranging from trace amounts in some species to 26.31 mg/g DW in the roots of *S. maximowicziana* Hemsl. [2]. However, its content in individual plants can be very variable. Previous reports indicated that the rosmarinic acid (RA) content of the aerial parts of only one species, *S. officinalis* ranged from 5.5 to 39.3 mg/g DW, depending on collection site and the extraction method [14,29]. Rosmarinic acid hexoside was found in similar amounts in the shoot and root extracts of *S. bulleyana* (*cir*. 0.3 mg/g DW) (Table 2). The compound has been previously found in comparable quantities in *S. bulleyana* roots [6].

The dominant compound in the roots of *S. bulleyana* was the caffeic acid trimer, salvianolic acid K (12.34 mg/g DW); this quantity was much higher than in the aerial parts (0.76 mg/g DW). Kasimu et al. recorded a much lower level of this compound in the roots (0.36 mg/g DW) [6], together with a high amount of salvianolic acid B (15.9 mg/g DW). In our study, significantly smaller amounts of salvianolic acid B isomer were found, i.e., less than 1 mg/g DW. This difference in the quantitative composition of the raw material could be caused by the significantly different growth conditions of the plants, which would stimulate the enzymes of a different pathway of the polyphenolic acid metabolism. It may be also conditioned by the genotype of a particular cultivar. Similar differences have been found for example for *S leriifolia* Benth.; the content of salvianolic acid B in the leaves (17.74 mg/g DW) and roots (1.75 mg/g DW) of one wild population of the species [29] was found to be much higher than in the leaves (0.13 mg/g DW) and roots (0.11 mg/g DW) of another wild population in another study [30]. This may indicate that salvianolic acid production in plants is highly variable, and its content in the same species might be affected by such factors as environmental growing conditions, season of harvest, or vegetation period. However, there is a problem in verifying this hypothesis due to the final stages of the metabolic pathways of individual more complex polyphenolic acids and their derivatives remaining poorly understood. 

Another significant phenolic derivative observed in shoot extracts of *S. bulleyana* was the lithospermic acid isomer, with a content of approximately 3.8 mg/g DW. Lithospermic acid itself was previously found in amounts ranging from 0.4 to 4.5 mg/g DW in *S. miltiorrhiza* and in the roots of several other species of Chinese sage used as Danshen [2]. Additionally, unlike Kasimu et al. [6], we estimated the root concentration of the isomer of salvianolic acid F to be 0.16 mg/g DW. It has also been reported in the roots of field-grown *S. viridis* at a concentration of 0.43 mg/g DW [19].

As the other polyphenolic acid derivatives found in *S. bulleyana* root and shoot extracts were present in much lower amounts, their combined polyphenolic potential was estimated as the sum of their caffeic acid equivalents. The total content of phenolic acid derivatives was almost twice as high in the roots (cir. 23 mg/g DW) as in the shoots of this species (14.5 mg/g DW).

The flavonoid compounds also constituted a proportion of the shoot extracts; however, no previous study included any qualitative assessment of these metabolites in *S. bulleyana* extracts. Our findings indicate that this group was dominated by luteolin, as well as various other flavones, estimated in the amount of 2.5 mg/g DW expressed in aglycone equivalents of these compounds obtained after the hydrolysis of the extract (Table 2). Therefore, the analyzed flavonoids were present in the shoot extract at much lower levels than the phenolic acids. Similar results have been recently reported on the phytochemical composition of the aerial parts of hydromethanolic/methanolic extracts of *S. viridis* and *S. verticillata* [7,14].

### 2.3. Antioxidant Properties 

**Figure 3:**
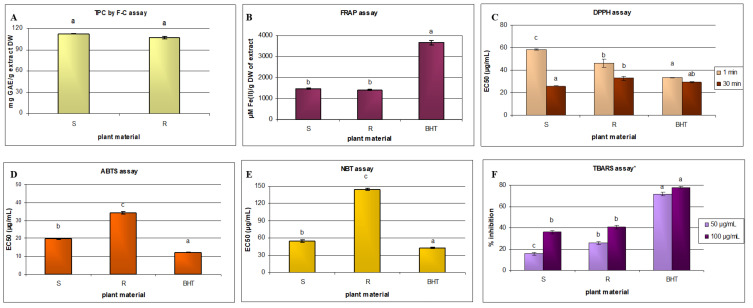
Antioxidant properties of the aerial parts and roots of *S. bulleyana* hydromethanolic extracts in TPC (**A**), FRAP (**B**), DPPH (**C**), ABTS (**D**), NBT (**E**) and TBARS (**F**) assays. Values are presented as means ± standard error (SE). Values marked with different letters for the same assay have statistically significant difference (*p* < 0.05). * The values are the percentage of inhibition for the given extract concentration.

Extracts rich in phenolics are known to counteract oxidative stress via several different mechanisms. Therefore, in the present study, the antioxidant potential of the *Salvia* extracts was evaluated by reducing power (FRAP), free radical scavenging (DPPH, ABTS, and O_2_*^•^*^−^), and inhibition of lipid peroxidation assays. The results are summarized in Figure 3, and both extracts showed significant antioxidant potential.

The reductive capacity of antioxidants can be determined based on electron transfer by the Folin–Ciocalteu total phenolic content (TPC) method. However, although this method has been widely applied in vitro, the resulting data cannot be extrapolated in a simple way to in vivo effects. Nevertheless, in clinical studies, the obtained results turned out to be inversely correlated with DNA oxidation, cardiovascular risk parameters, and aging [31]. Due to the prevalence of this method, the obtained total polyphenol content could be easily compared with that of other sage species [10,32,33]. The extract from *S. bulleyana* aerial parts showed only a slightly higher TPC value than the root extract: 112.5 mg GAE/g of extract DW, and 106.9 mg GAE/g of extract DW (*p* > 0.05). This can be attributed to the fact that polyphenolic acids are also accompanied by flavonoids in the shoots. In contrast, the HPLC analysis identified a significantly higher total polyphenol content in the roots than in the shoots (Figure 3A). This difference might come from the fact that the Folin–Ciocalteu method does not measure the actual content of phenolic compounds, but the overall antioxidant potential of the sample. It also indicates a significant contribution of flavonoids (present in the shoot extract in low concentrations) to this activity.

The shoots and roots of *Salvia* species often demonstrate different TPC values; for example, the aerial parts of *S. fruticosa* Mill. or *S. przewalskii* have been found to have a higher TPC than the roots [33,34], while the roots of *S. officinalis* have a higher TPC than the shoots [32]. The aerial parts of *S. bulleyana* were found to contain a similar TPC to *S. euphratica* (108 mg GAE/g extract), *S. verticillata* (116.23 mg GAE/g extract) [10], and *S. viridis* (106 mg GAE/g extract) [7], but a lower TPC than the aerial parts of *S. nemorosa* L. (294.9 mg GAE/g extract) [35]. Regarding the root extracts, our results obtained for S. *bulleyana* roots were similar to those for *S. viridis* root obtained by the same extraction method (102.03 mg GAE/g extract) [11], but significantly higher than for *S. fruticosa* (80 mg GAE/g extract) [34] and *S. officinalis* (62 mg GAE/g extract) [32]. In addition, our findings were significantly higher than for *S. miltiorrhiza* root (40.1 mg GAE/g extract) [33]. The superiority of the investigated roots, to the roots of the latter species, is of particular interest due to the use of *S. bulleyana* as a counterpart to Danshen in Chinese medicine. Since *S. bulleyana* roots have recently been found to contain lower levels of tanshinones than *S. miltiorrhiza* roots [5], the higher levels of phenolics might be an alternative justification for the use of *S. bulleyana* in therapies. 

FRAP assay is often used to evaluate the antioxidant potential of plant extracts, such as those of herbs, fruits, and vegetables, by determining their total reduction capacity. The results indicated that the aerial parts and roots of *S. bulleyana* demonstrated a similar antioxidant potential (Figure 3B), which is most likely correlated with the TPC. A similar relationship was found between the Folin–Ciocalteu total phenolic content and FRAP assays for other sage species [7,19].

The antiradical capacities of the extracts, evaluated after one minute and 30 min using the DPPH assay, are shown in Figure 3C as EC_50_ values. At 30 min, i.e., when the reaction was completed, the shoots demonstrated a significantly higher DPPH activity (25.9 µg/mL) than the roots (32.9 µg/mL); however, at the initial reading, a lower EC_50_ value was observed for the root extract, indicating more rapid DPPH radical binding. The DPPH test is commonly used for assessing the antioxidant potential of sage species, and our findings for *S bulleyana* shoots were comparable to those other sages with strong antioxidant activity, such as extracts of aerial parts of *S. verticillata* (33.4 μg/mL) [14], *S. officinalis* (23.2 μg/mL) [32], and *S. cadmica* (34.91 μg/mL) [20], or shoots of *S. elegans* (10.7 μg/mL) and *S. greggii* (21.1 μg/mL) [15].

The *S. bulleyana* root extract also demonstrated a strong antiradical activity, comparable to other species such as *S. officinalis* (32.5 μg/mL) [32]. It is also worth emphasizing that after 30 min, both (root and shoot) extracts demonstrated similar DPPH radical scavenging capacity to the BHT used as a positive control (Figure 3C). Similarly, the shoots of *S. bulleyana* demonstrated greater radical scavenging ability in a ABTS test (19.9 μg/mL) than the roots (34.3 μg/mL) (Figure 3D); however, both extracts gave stronger results than the methanolic extract of the aerial parts of *S. verticillata* (67.01 μg/mL) [14].

DPPH and ABTS scavenging assays are often used to verify the antioxidant potential of plant materials, due to their simplicity, speed of reaction, and low cost. However, in the present study, the reducing properties towards other reactive particles, such as O_2_*^•^*^−^, which form under natural conditions in living organisms, were also evaluated. The presence of excess levels of these radicals, associated with oxidative stress, is considered an important factor in civilization diseases, such as cancer, cardiovascular diseases, atherosclerosis, diabetes, neurodegenerative diseases, and accelerated aging [1].

In this test, the hydromethanolic shoot extract of *S. bulleyana* showed almost three times greater activity than that of the roots (Figure 3E); in addition, the aerial parts of *S. bulleyana* (54.6 μg/mL) demonstrated a similar scavenging potential as that of *S. viridis* (83 μg/mL) and *S. greggii* (61.7 μg/mL), and slightly higher than that *S. officinalis* (32.8 μg/mL) [7,15]. However, in contrast to the DPPH findings, even the much more active shoot extract showed significantly weaker activity than the positive control (Figure 3E). This is not surprising, as it is known that different antioxidants may respond differently to different radical or oxidant sources, which could be related to the different mechanisms of action. For example, compared to phenolics, carotenoid plant pigments are more effective at scavenging singlet oxygen, but less effective for peroxyl radicals [36]. Although the superoxide anion is not very active, it is important to control its level because it ultimately stimulates the formation of strong hydroxyl radicals and singlet oxygen. 

Interestingly, the *S. bulleyana* root extract inhibited lipid peroxidation at both 50 μg and 100 μg, more strongly than the shoot extract, 26% compared to 16% at 50 μg, and 41% compared to 36% at 100 μg (Figure 3F). This is an important effect, as it indicates a potential to counteract oxidative lipid degradation occurring in the human body, or in food and cosmetic products during their storage. However, stronger lipid peroxidation was observed for shoot extracts of *S. officinalis* and *S. viridis* at a concentration of 100 μg [7,32]. 

The polyphenolic compounds present in the *S. bulleyana* extracts are undoubtedly responsible for their antioxidant properties, but at this stage of the research it is difficult to unambiguously correlate the obtained results of biological studies with the phytochemical profile of the raw material. One of the compounds that might be greatly involved in this activity is RA, the dominant component of the extracts, and present at similar levels in shoots and roots. Previous studies have demonstrated the significant relationship between RA content and antioxidant activity of some Lamiaceae plants [21,37]. The strong antiradical potential of pure rosmarinic acid in a DPPH assay has also been reported; IC_50_ = 12.4 µg/mL [38]. An antioxidant activity was also documented for lithospermic acid [39], although it is not clear whether the same strong properties could be found for the isomer of this compound detected in the *S. bulleyana* shoot extract, due to the lack of knowledge of its detailed structure. So far, there are no available literature data that have documented the activity of salvianolic acid K compound, found in high level in *S. bulleyana* roots. However, like for other salvianolic acids, e.g., salvianolic acid B, which is regarded as the main antioxidant of *S. miltiorhhiza* [40], such properties could be expected. On the other hand, the roots and shoots of *S. bulleyana* demonstrated similar antioxidant activity, despite the higher total content of phenolic compounds in roots than in shoots. This may indicate a significant contribution of flavonoids, which were detected only in the aerial parts of the plant. This is not surprising since numerous studies have reported remarkable antioxidant properties of flavones, especially luteolin and its derivatives [41]. However, it should be remembered that antioxidant activity depends on the detailed chemical structure of a compound, and only after isolation can its potential be fully assessed. Moreover, in case of complex matrices, such as plant extracts, the interactions between constituents (e.g., synergistic) are an additional aspect that should be taken into account.

Although the antioxidant activities of the analyzed *S. bulleyana* extracts were generally not as strong as those recorded for standard antioxidants, such as BHT (Figure 3), our findings do nevertheless indicate that the raw material may have significant therapeutic potential. Moreover, plant metabolites do not usually show the side effects that are often shown by synthetic compounds. Additionally, it should be remembered that the analyzed plant extracts contained a lot of ballast compounds, and the individual pure working ingredients formed only a part of them. This creates further opportunities for the purification and concentration of the extracts, to prepare products with increased activity. 

## 3. Materials and Methods

### 3.1. The Origin of the Plant Material

The roots and shoots of 2 year-old *Salvia bulleyana* plants were collected in late July and early August 2019, i.e., during the flowering stage, from the garden of the Department of Pharmacognosy, Medical University of Lodz (51°77′ N, 19°49′ E) (central Poland, Europe) (Figure 1). The seeds from which the plants were initiated were derived from the botanical garden of the University of Bonn (Germany). The plant’s botanical identification was performed on the basis of the Flora of China [42]. Voucher specimens (IG/SBRF/2019 and IG/SBSF/2019) were deposited at the Department of Biology and Pharmaceutical Botany, Medical University of Lodz (Lodz, Poland).

### 3.2. Standards and Reagents

HPLC grade methanol and acetonitrile and Folin–Ciocalteau’s reagent were supplied from POCh (Gliwice, Poland). Nitrotetrazolinum blue chloride (NBT), phenazine methosulfate, NADH, 2,2-diphenyl-1-pikryl-hydrazyl (DPPH), potassium persulfate, 2,2′-azobis(2-amidinopropane)dihydrochloride (AAPH), 2,2-azino-bis (3-ethylbenzothiazoline-6-sulfonic acid) diammonium salt (ABTS), FeCl_3_, 2,4,6-Tris(2-pyridyl)-s-triazine (TPTZ), thiobarbituric acid (TBA), and linoleic acid were purchased from Sigma-Aldrich/Merck (Darmstadt, Germany). Butylated hydroxytoluene (BHT) was supplied from Supleco (Bellefonte, PA, USA). HPLC grade reference standards of salvianolic acid A and salvianolic acid F were supplied from ChemFaces (Hubei, China), caffeic acid and luteolin from Sigma Aldrich/Merck (Darmstadt, Germany), and salvianolic acid B and rosmarinic acid (RA) from Extrasynthese (Genay, France).

### 3.3. Extraction Procedure

The collected plant materials (aerial and underground parts separately) were dried in the dark at room temperature. Immediately before extraction, they were ground to powder using a laboratory mill. The powdered plant material was pre-extracted overnight with 30 mL chloroform in a rotary shaker (70 rpm) at room temperature, and then filtered; the chloroform extract was discarded. 

Briefly, 500 mg of plant material was extracted (using a UD-20 ultrasonic disintegrator) three times with 30 mL of 80% methanol at 40 °C for 15 min for use in qualitative analysis, in polyphenolic acid qualitative analysis, and antioxidant assays [7]. The extracts were combined and evaporated under reduced pressure, and the dry extracts were stored in a refrigerator at 2–4 °C. Before analyses, the extracts were dissolved in 80% methanol. 

For the analysis of flavonoid content, acidic hydrolysis of the native glycosides was performed. Briefly, 500 mg of the plant material were refluxed for 1 h in a mixture of 6 mL of 25% hydrochloric acid and 20 mL of 80% aqueous methanol. After filtration, the plant material was extracted twice for 20 min with 20 mL of 80% methanol and filtered. The obtained extracts were combined and diluted with the solvent to 50 mL in a measuring flask. 

### 3.4. Qualitative UHPLC-PDA-ESI-MS Analysis

Before analysis, the dry extracts were dissolved in 5 mL of 0.1% HCOOH in 80% methanol and filtered through a 0.45 µm Chromafil membrane (Machery-Nagel, Duren, Germany). Phenolic compounds in the samples were identified using an UPLC-3000 RS system (Dionex, Germany) equipped with an AmaZon SL ion trap mass spectrometer, with an ESI interface (Bruker Daltonik GmbH, Bremen, Germany) and a PDF detector. Separation was performed on a Zorbax SB-C18 column (150 × 2.1 mm, 1.9 μm) (Agilent, Santa Clara, CA, USA). The mobile phase consisted of 0.1% HCOOH in water (A) and 0.1% HCOOH in acetonitrile (B), using the following gradients: 0–60 min, 5–40% B. The optimized conditions of the MS detector were set as follows: nebulizer pressure of 40 psi; drying gas flow rate of 9 L/min; nitrogen gas temperature of 300 °C; and a capillary voltage of 4.5 kV. The analysis was carried out using a scan from 100 to 2200 *m/z*. UV spectra were recorded in the range of 200–400 nm, and chromatograms were acquired at 325 nm. The compounds were analyzed in negative ion mode, and identified by comparing the data on their retention time, UV–vis, and mass spectra with the data obtained for standards, or the data reported in the literature [7,8,9,10,11,12,13].

### 3.5. Quantitative HPLC-PDA Analysis of Polyphenolic Acids

Before the analysis, extracts were dissolved in 80% methanol and filtered through a PTFE syringe filter (25 mm, 0.22 μm). Compounds were analyzed using an Elite LaChrom Hitachi system according to the method described by Wojciechowska et al. [13]. The tentatively identified peaks were quantified relatively, using calibration curves of closely related authentic standards: rosmarinic acid for quantification of rosmarinic acid hexoside and methylrosmarinate; salvianolic acid B and salvianolic acid F for quantification of salvianolic acid B isomer and salvianolic F isomer, respectively; salvianolic acid A for quantification of salvianolic acid K and lithospermic acid isomer. Moreover, other polyphenolic acid derivatives and phenylethanoids, which were present in the extracts in small amounts, and whose content was given as the sum of other polyphenolic compounds, were quantified against caffeic acid. The amounts of compound in the plant material were expressed as mg/g DW of plant material.

### 3.6. Quantitative HPLC-PDA Analysis of Flavonoids

The hydrolyzed extracts were filtered through a PTFE syringe filter (25 mm, 0.22 µm) and analyzed using an Elite LaChrom Hitachi system. The separation was carried out on a C18 Ascentis^®^ Express column (2.7 μm, 150 mm × 4.6 mm i.d.; Supelco, Bellefonte, PA, USA) with a C18 Ascentis^®^ C18 Supelguard guard column (3 μm, 20 mm × 4 mm i.d.; Supelco) using a mobile phase, consisting of solvent A (0.5% orthophosphoric acid, *v/v*) and solvent B (acetonitrile). The elution gradient was as follows: 0–1 min, 15% B (*v*/*v*); 1–20 min 15–25% B; 20–21 min, 25–75% B; 21–25 min, 75% B; 25–30 min, 15% B. The flow rate was 1.4 mL/min, and the temperature of the analysis 35 °C. UV–vis spectra were recorded over the range of 200–600 nm. Detected flavonoid aglycones were quantified (*λ* = 345 nm) based on the calibration curve of luteolin, and their content was expressed in mg/g DW.

### 3.7. Antioxidant Assays

#### 3.7.1. Total Phenolic Content

The total phenolic (TPC) content of *Salvia* extracts was determined according to the adapted Folin–Ciocalteu colorimetric method, as described earlier by Grzegorczyk-Karolak et al. [43]. The absorbance of the obtained blue solution was measured at 765 nm using a Ray Leigh UV-1601 spectrophotometer (Beijing Reyleigh Corp., Beijing, China). The results were expressed as mg of gallic equivalents (GAE) per g of dry extracts.

#### 3.7.2. FRAP Assay

The ferric reducing potential of the extract was determined by FRAP assay as described by Grzegorczyk-Karolak et al. [44]. After mixing, the hydromethanolic extract, water, and FRAP reagent were kept in a water bath for 30 min at 37 °C. After cooling to room temperature, the absorbance of the extracts was read at 595 nm, and the results were expressed in µM Fe (II)/g of dry extract.

#### 3.7.3. DPPH Radical Scavenging Assay

To determine DPPH radical scavenging activity, 2 mL of extract at different concentrations (2, 10, 20, 100, 200, 400, and 800 μg/mL) was mixed with 2 mL of DPPH solution, as detailed by Grzegorczyk-Karolak and Kiss [7]. After 1 min and 30 min of incubation at room temperature, the absorbance was measured at 517 nm. The results were expressed as EC_50_ value [μg/mL], i.e., the concentration of extract that reduces the initial radical concentration by 50%.

#### 3.7.4. ABTS Radical Scavenging Assay

The ABTS scavenging properties were measured based on the method of Grzegorczyk-Karolak et al. [44]. Briefly, 2 mL of extracts at different concentrations (between 2–400 μg/mL) were mixed with the 2 mL of freshly-prepared ABTS solution. After 10 min of incubation at 25 °C, in the dark, the absorbance of the solutions was measured at 735 nm. The results of the ABTS scavenging test were expressed as EC_50_ [μg/mL], defined as the concentration of the extract required to reduce the radical concentration by 50%.

#### 3.7.5. O_2_^•−^ Scavenging Assay

The superoxide radical (O_2_*^•^*^−^) scavenging capacity was measured based on the reduction of NBT (nitroblue tetrazolinum) as described by Grzegorczyk-Karolak and Kiss [7]. The absorbance of the reaction mixture after a five-minute incubation at room temperature was measured at 560 nm. As in previous radical tests, the results were expressed as EC_50_ value [μg/mL], i.e., the concentration of extract needed to reduce the initial radical concentration by 50%.

#### 3.7.6. Inhibition of Linoleic Acid Peroxidation Assay

The antioxidant activity of *S. bulleyana* extract was determined by inhibition of lipid peroxidation by TBARS (thiobarbituric acid reactive substances) assay. Peroxidation of linoleic acid was induced using AAPH solution, and the analytical procedure was performed as described by Grzegorczyk-Karolak and Kiss [7], with plant extracts added to the reaction mixture at a concentration of 50 μg/mL or 100 μg/mL. The absorbance was measured at 532 nm. The inhibition percent of linoleic acid peroxidation was calculated using the following equation:

% inhibition = [(Abs control − Abs sample − Abs extract)/(Abs control − Abs extract)] × 100

where Abs control is the absorbance of the mixture with methanol instead of an extract, and Abs extract is the absorbance of the mixture with methanol instead of linoleic acid.

### 3.8. Statistical Analysis

Data were reported as means ±SE (standard error) of three independent tests. All experiments were repeated three times. The means were compared using the one-way ANOVA test and multivariate analysis of variance followed by the Tukey’s post hoc test (*p* < 0.05). The statistical analysis was conducted with Statistica 13.1 PL for Windows (StatSoft Inc., Kraków, Poland).

## 4. Conclusions

In the present study, identification of phenolic compounds in the hydromethanolic extracts of aerial parts and underground parts of *S. bulleyana* was carried out. This is the first detailed phytochemical analysis of raw materials derived from this species. In summary, the extracts of the root parts contain phenolic acid derivatives, while those of the shoot parts contain phenolic acid derivatives and flavonoids. However, the chemical composition of both raw materials is significantly different from that specified for Danshen.

According to our study, both extracts of *S. bulleyana* were shown to be promising antioxidant agents. The activity of the analyzed extracts was beneficial compared to the other *Salvia* species, which indicates the possibility of using this species interchangeably with others used all over the world in official medicine. The proved antioxidant properties of the studied extracts indicate their potential as alternative ingredients for functional products supporting the management of chronic complications, such as cardiovascular disease, diabetes mellitus, or cancer. This activity could be useful, not only for the prevention of civilization diseases, but also in the food industry for extending the shelf-life of food products by preventing lipid oxidation, or as antioxidants protecting cosmetic formulations against aging. Although estimated activities were correlated with the levels of polyphenols in the studied extracts, flavonoids seemed to have a higher impact on this activity. Our findings could serve as a base for future research on this species, which is necessary to understand the mechanisms of the in vivo activities of the raw material.

## Figures and Tables

**Table 1:** Characterization of compounds from aerial parts and roots of *Salvia bulleyana* hydromethanolic extracts determined by ultrahigh-performance liquid chromatography with electrospray ionization mass spectrometry (UHPLC-MS/MS).

Peak No.	R_t_ [min]	λ_max_ (nm)	[M−H]^−^	Fragmentation Ions	Tentative Identification	Reference	Plant Material
1	2.7		191		Quinic acid	[15]	S
2	12.6	295sh, 342	297	179, 161, **135**	Caffeoyl-threonic acid (I)	[9]	S
3	14.5	297sh, 326	297	179, 161, **135**	Caffeoyl-threonic acid (II)	[9]	S
4	18.6	292sh, 321	179	**135**	Caffeic acid	[19]	R, S
5	19.8	328	385	**223**, 247, 205, 164	Sinapic acid hexose	[11]	S
6	20.1	291sh, 325	297	279, 179, **135**	Caffeoyl-threonic acid (III)	[9]	S
7	21.6	267, 340	801	**639**, 477, 463, 301	**Hydroxyluteolin-*O*-dihexoside-*O*-hexuronide**	[12]	S
8	22.3	279, 323	489	**223**, 205	Sinapic acid derivative		S
9	23.1	277, 323	706	662, 619, 526, 508, 482, 464, 439, **420**, 376, 253, 197	Unknown compound		S
10	26.6	281, 324	571	553, 527, 509, 483, **439**, 285, 197, 179	Yunnaneic acid E	[12]	S
11	28.1	270, 336	639	**477**, 301	Hydroxyluteolin-*O*-hexoside-*O*-hexuronide	[12]	S
12	28.5	272, 323	623	**461**, 447, 285	Luteolin-*O*-hexoside-*O*-hexuronide	[8]	S
13	29.3	267, 343	593	**285**	Luteolin-*O*-rhamnohexoside	[7]	S
14	30.8	269, 340	461	**285**	Luteolin-*O*-hexuronide	[11]	S
15	31.5	287sh, 319	521	359	Rosmarinic acid hexoside	[19]	R, S
16	32.9	269, 328	555	537, 511, 449, **357**, 313, 269, 241	Salvianolic acid K isomer	[20]	S
17	32.9	269, 328	577	**269**	Trihydroxyflavone-*O*-rhamnohexoside		S
18	33.5	271, 331	607	**299**	Trihydroxymethoxyflavone-*O*-rhamnohexoside	[8,12]	S
19	35.0	268, 334	445	**269**, 175	Trihydroxyflavone-*O*-hexuronide		S
20	36.6	289sh, 325	359	223, 197, 179, **161**	Rosmarinic acid	[19]	R, S
21	37.4	286, 321	555	537, **493**, 449, 359, 313, 269	Salvianolic acid K	[13]	R, S
22	38.6	284sh, 324	683	521, **485**, 359, 321	Caffeic acid derivative		S
23	40.2	289sh, 324	537	**493**, 359	Lithospermic acid isomer (I)	[20]	S
24	40.7	286, 328	651	505, **475**, 329, 265, 193	Martinoside	[7]	S
25	41.4	282, 318	343	325, 223, 197, **179**, 135	Dehydrorosmarinic acid	[21]	S
26	41.9		717	**519**, 321	Salvianolic acid B isomer	[21]	R
27	43.0	285, 324	727	529, **359**	Rosmarinic acid sinapoyl-hexoside (I)	[22]	S
28	43.5	286sh, 327	373	179, 135	Methyl rosmarinate	[19]	R
29	45.2	281, 324	537	**493**, 359, 295	Lithospermic acid isomer II	[20]	S
30	45.6	280, 321	727	**565**, 521, 359	Rosmarinic acid sinapoyl-hexoside (II)	[22]	S
31	46.1	278, 320	727	565, 529, **359**	Rosmarinic acid sinapoyl-hexoside (III)	[22]	S
32	46.8	282, 325	693	**651**, 635, 475,	Acetylmartynoside		S
33	47.5	273sh, 313	811	**635**, 443, 285	Unidentified flavone		S
34	48.8	285sh, 324	551	**519**, 359, 339, 161	Monomethyl lithospermate isomer	[23]	S
35	50.8	336	313	269,161	Salvianolic acid F isomer (I)	[13]	R
36	51.2	284, 319	717	**519**, 359, 339	Salvianolic acid B isomer (II)		S
37	53.6	302sh, 335	313	269, 203, 161	Salvianolic acid F isomer (II)	[13]	R
38	55.8	278sh, 327	735	693, 547, 505	Diacetylmartynoside	[24]	R, S

S-shoots, R-roots. The dominant fragmentation ions are highlighted in bold.

**Table 2:** Phenolic compound content (mg/g DW) in aerial parts and roots of *S. bulleyana* hydromethanolic extracts determined by high-performance liquid chromatography with photodiode (HPLC-PDA).

Compound	Aerial Parts	Roots
Caffeic acid	0.03 ± 0.01 ^b^	0.24 ± 0.007 ^a^
Rosmarinic acid hexoside	0.25 ± 0.05 ^a^	0.30 ± 0.004 ^a^
Rosmarinic acid	6.07 ± 0.73 ^b^	8.34 ± 0.14 ^a^
Salvianolic acid K	0.76 ± 0.15 ^b^	12.34 ± 0.05 ^a^
Lithospermic acid isomer (I)	3.78 ± 0.51	nd
Salvianolic acid B isomer (I)	nd	0.923 ± 0.006
Methyl rosmarinate	nd	0.58 ± 0.012
Salvianolic acid F isomer (II)	nd	0.156 ± 0.004
Sum of other PAD	1.08 ± 0.46 ^a^	0.206 ± 0.008 ^b^
TF	2.42 ± 0.11	nd
PAD	14.39 ± 2.02 ^b^	23.085 ± 0.23 ^a^

Values are presented as means ± standard error (SE). Values marked with different letters for the same metabolite different statistically significant (*p* < 0.05). PAD: phenolic acid derivatives. TF: total content of flavonoid aglycones after hydrolysis.
